# Case of Placenta Prævia

**Published:** 1845-11

**Authors:** J. C. Parker

**Affiliations:** Surgeon to the Bridgewater Infirmary, &c.


*Case of Placenta Prævia.* By J. C. Parker, Surgeon to the Bridgwater Infirmary, &c.—The great importance of establishing a proper rule of practice in these important cases—placenta prævia—is so evident, that no apology is necessary for offering the following case to your readers:—

I was sent for a few years since, to visit a Mrs. Morris, of Westonzoyland, near Bridgwater, by a surgeon who was in attendance on her in her confinement for her second child. On my arrival I learnt that she had been suffering from hæmorrhage about a fortnight previous, which, after continuing a short time, had suddenly ceased, that it had again occurred about four hours before my arrival, and had ceased after continuing about an hour, accompanied by active labour pains.

On making an examination, to my astonishment I found the placenta lying on the bed, completely expelled from the vagina, and about two inches of the umbilical cord also protruded with it; the arm of a full-grown fœtus was also presenting and protruding from the vagina as far as it could. There was not the slightest hæmorrhage; there were regular pains at short intervals; the patient complained of feeling faint and weak; she had a small quick pulse. After giving her a full opiate, in a little warm brandy and water, I slowly and cautiously introduced my hand, got one foot, and with little comparative difficulty turned; the delivery was soon effected, there was not the slightest hæmorrhage, and the patient recovered without one unfavourable symptom.

Here it appears to me nature effected that which, in cases of placenta prævia, art should imitate, and after the many important cases published in your Journal, and the able investigations conducted by Drs. Radford and Simpson on this subject, the best rule of practice appears established.—*Prov. Med. and Surg. Journal.*

